# Fast and scalable neural embedding models for biomedical sentence classification

**DOI:** 10.1186/s12859-018-2496-4

**Published:** 2018-12-22

**Authors:** Asan Agibetov, Kathrin Blagec, Hong Xu, Matthias Samwald

**Affiliations:** Section for Artificial Intelligence and Decision Support, Medical University of Vienna, Währinger Strasse 25A, OG1, Vienna, 1090 Austria

**Keywords:** Natural language processing, Text classification, Neural networks, Word vector models, FastText, Scientific abstracts

## Abstract

**Background:**

Biomedical literature is expanding rapidly, and tools that help locate information of interest are needed. To this end, a multitude of different approaches for classifying sentences in biomedical publications according to their coarse semantic and rhetoric categories (e.g., Background, Methods, Results, Conclusions) have been devised, with recent state-of-the-art results reported for a complex deep learning model. Recent evidence showed that shallow and wide neural models such as fastText can provide results that are competitive or superior to complex deep learning models while requiring drastically lower training times and having better scalability. We analyze the efficacy of the fastText model in the classification of biomedical sentences in the PubMed 200k RCT benchmark, and introduce a simple pre-processing step that enables the application of fastText on sentence sequences. Furthermore, we explore the utility of two unsupervised pre-training approaches in scenarios where labeled training data are limited.

**Results:**

Our fastText-based methodology yields a state-of-the-art F1 score of.917 on the PubMed 200k benchmark when sentence ordering is taken into account, with a training time of only 73 s on standard hardware. Applying fastText on single sentences, without taking sentence ordering into account, yielded an F1 score of.852 (training time 13 s). Unsupervised pre-training of N-gram vectors greatly improved the results for small training set sizes, with an increase of F1 score of.21 to.74 when trained on only 1000 randomly picked sentences without taking sentence ordering into account.

**Conclusions:**

Because of it’s ease of use and performance, fastText should be among the first choices of tools when tackling biomedical text classification problems with large corpora. Unsupervised pre-training of N-gram vectors on domain-specific corpora also makes it possible to apply fastText when labeled training data are limited.

## Background

Biomedical literature is vast and rapidly expanding. With over 27 million articles currently in PubMed, it is increasingly difficult for researchers and healthcare professionals to efficiently search, extract and synthesize knowledge from diverse publications. Technological solutions that help users locate text snippets of interest in a quickly and highly targeted manner are needed. To this end, a multitude of different approaches for classifying sentences in PubMed abstracts according to their coarse semantic and rhetoric categories (e.g., Introduction/Background, Methods, Results, Conclusions) has been devised. Many different methodological approaches to this task have been described in literature, including naive Bayes [1–4], support vector machines [2, 3, 5], Hidden Markov models [6], Conditional Random fields (CRFs) [7–9], and advanced, semi-automatic engineering of features [10].

Recently, a new state-of-the-art methodology for the task of sequential sentence categorization in PubMed abstracts based on a deep learning model has been reported [11]. The model is based on a sophisticated architecture with bi-directional Long short-term memory (LSTM) layers applied to characters and word tokens, taking sentence ordering in the abstract into account. The authors demonstrate superior results of this deep model on the established NICTA-PIBOSO corpus [9, 11], as well as the newly created, larger PubMed 200k RCT benchmark dataset [12].

Training deep neural networks on large text data is often not trivial, since they require careful hyperparameter optimization to provide good results, require the use of graphics processor units (GPUs) for performant training, and often take a long time to train. Recent evidence showed that shallow and wide neural models such as the fastText model [13] based on token embeddings can provide results that are competitive with complex deep learning models while requiring drastically lower training times and having better scalability [13, 14] without necessitating the utilization of GPUs.

In this work, we analyze the applicability of the fastText model on the classification of biomedical sentences in the PubMed 200k RCT benchmark. Specifically, we demonstrate how simple corpus preprocessing can be used to train fastText on sentence sequences instead of singular sentences, and how such an approach can yield state-of-the-art results while retaining very short training times. Furthermore, we explore approaches of semi-supervised learning, where models are pre-trained through unsupervised training on predicting word contexts or sentence reconstruction tasks, and demonstrate that unsupervised pre-training greatly improves classification quality when the labeled training data are limited.

## Methods

### Datasets

PubMed 200k RCT is a new dataset derived from PubMed that is intended as a benchmark for sequential sentence classification. It is made up of two subsets, PubMed 200k and PubMed 20k. PubMed 200k contains approximately 200,000 abstracts of randomized controlled trials (RCTs), with a total of 2,2 million sentences. PubMed 20k is a smaller version of the PubMed 200k dataset, containing only 20,000 abstracts. Each sentence in the datasets is labeled with one of five labels (‘objective’, ‘background’, ‘method’, ‘result’ or ‘conclusion’). The datasets are divided into predefined training, validation and test data splits. Details about the construction of the datasets and dataset statistics can be found in [11].

To investigate if additional and more diverse training data could further improve classification results, we also created an extended corpus, where the training data of the PubMed 200k dataset were augmented with additional structured abstracts derived from journals with a medical focus indexed by PubMed. The training split of this extended corpus contains 872,000 abstracts (compared to only 190,000 abstracts in the training split of PubMed 200k). Validation and test data for the extended corpus remained the same as for the PubMed 200k dataset.

All corpora were lower-cased and punctuation was separated from words through added whitespace. Statistics of all datasets are summarized in Table 1.

**Table 1:** $|\mathcal {V}|$ denotes vocabulary size. For train, validation and test datasets, the number of abstracts followed and the number of sentences (in parentheses) are shown

Dataset	$|\mathcal {V}|$	Train	Validation	Test
PubMed 20k	68k	15k (180k)	2.5k (30k)	2.5k (30k)
PubMed 200k	331k	190k (2.2M)	2.5k (29k)	2.5k (29k)
Extended corpus	451k	872k (10,3M)	2.5k (29k)	2.5k (29k)

### Neural embedding models for n-gram embeddings

**Fig. 1:**
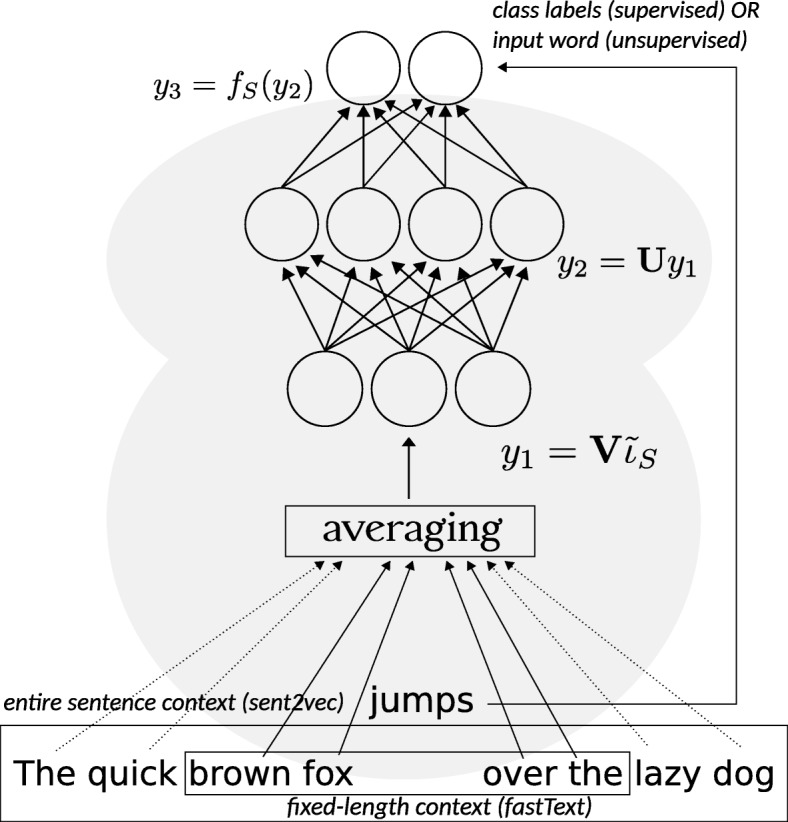
Schematic representation of the neural embedding model for sentences (supervised and unsupervised) consisting of two embedding layers and a final softmax layer over *k* classes (for the supervised case). In the unsupervised case $k=|\mathcal {V}|$ and the softmax outputs the probability of the target word (over all vocabulary, as in C-BOW model) given its context: fixed-length context for fastText and entire sentence context for sent2vec. Independently of the training mode (e.g., supervised vs unsupervised) word embeddings are stored as columns in the weight matrix *V* of the first embedding layer. Note that in the unsupervised case the rows of the weight matrix *U* of the second embedding layer represent the embeddings for the “negative” words; these embeddings however are not used for the downstream machine learning tasks. In all instances the averaging of embeddings of constituent tokens ($\mathcal {\hat {\iota }}_{S}$) is performed by fastText (sent2vec implementation is based on fastText)

Recently introduced neural embedding word vector models are based on the so-called matrix factor models (sometimes also referred to as bi-linear models). The models are flexible as they can be easily adapted to both supervised and unsupervised learning, and can also be extended to n-gram embedding problems by simply creating additional embeddings for encountered n-grams. Embedding vectors are learned through a shallow neural network with a single hidden layer. These embedding vectors are learned implicitly, and collected in the weight matrix of the hidden layer (Fig. 1).

To simplify the notation, we present below the most general formalized form of these models, adapted from [15]:

1 $$\begin{array}{*{20}l} \underset{\mathbf{U}, \mathbf{V}}{\mathrm{arg\,min}} \sum\limits_{S \in C} f_{S}(\mathbf{UV} \mathbf{\iota}_{S}). \end{array} $$

The goal of this optimization problem is to find the parameter matrices $\mathbf {U} \in \mathbb {R}^{k \times h}, \mathbf {V} \in \mathbb {R}^{h \times |\mathcal {V}|}$ ($\mathcal {V}$ - vocabulary, set of all words or n-grams) that minimize (1). Learned embedding vectors will have dimension *h* and will be stored as columns in the matrix **V**. *S* are either the fixed-length context windows in the corpus $\mathcal {C}$ in the continuous bag-of-words (CBOW) model [16, 17], or entire sentences in the sent2vec model. $\mathbf {\iota }_{S} \in {0, 1}^{|\mathcal {V}|}$ (binary indicator vector) is the bag-of-words encoding of *S*. In the case of supervised learning $k \ll |\mathcal {V}|$ is the number of class labels, analogously $k = |\mathcal {V}|$ in the unsupervised case. The cost functions $f_{s} : \mathbb {R}^{k} \mapsto \mathbb {R}$ only depend on a single row of its input matrix (as a result of **UV**), and will have different forms depending on the learning task (we will detail this aspect below).

#### Supervised versus unsupervised training

In the following sections we describe the application of fully supervised learning as well as a mix of unsupervised learning followed by supervised learning. In the fully supervised training setup, a completely uninitialized embedding model was trained to predict labels and the resulting model was evaluated. In mixed setups, an unsupervised pre-training step was used to generate models that would then be used as the basis for supervised training in a second step. In unsupervised learning, the model was trained to predict the internal structure of the text, without requiring explicit labels (i.e., ‘without supervision’). Such unsupervised pre-training can induce useful representations of the content of sentences so that downstream supervised classification can potentially succeed with fewer training examples.

#### Supervised n-gram model of fastText

We used the fastText natural language processing library for sentence classification of biomedical text [13]. The methodology for sentence classification relies on the supervised n-gram classification model from fastText, where each sentence embedding is learned iteratively, based on the n-grams that appear in it. To this end each sentence is represented as a normalized bag of n-grams that capture the local word order. The fastText model can be seen as a shallow neural network that derives its capabilities by scaling up the number of learnable vector embeddings of n-gram features that are fed into the network. By adapting straightforwardly (1), we can represent this supervised classification model as a minimization of the negative log-likelihood over the class labels problem, as shown below:

2 $$  \underset{\mathbf{U}, \mathbf{V}}{\mathrm{arg\,min}} -\frac{1}{N} \sum\limits_{S \in C} y_{S} \log (f_{S}(\mathbf{UV} \tilde{\mathbf{\iota}}_{S})).  $$

We note that *y*_*S*_ is the label of the fixed-length context *S*, $k \ll |\mathcal {V}|$ is the number of class labels, and $\tilde {\iota _{S}}$ is the normalized bag of features encoding of *S* (i.e., ${\sum \nolimits }_{x \in \tilde {\iota _{S}}} x = 1$). Despite the simplicity of the model, it was shown to be competitive or superior to many deep neural architectures in text classification tasks [13], but also other tasks such as knowledge-base completion [14], with training times and resource requirements that are of often superior by orders of magnitude.

The fastText library was downloaded from GitHub (November 14, 2017) and compiled. For each experiment, an exhaustive hyperparameter grid search was conducted, hyperparameters considered were dimensionality of the n-gram embedding (10, 20 and 50 dimensions), word N-gram sizes (1, 2, 3 or 4 words), and number of training epochs (between 1 and 8 training epochs for 20k datasets and between 1 and 4 training epochs for larger datasets). All other hyperparameters were left at their default settings. All experiments were run on a machine with the Ubuntu 16.04 operating system, Intel Core i7-6700 4x3.40 Ghz processor and 32 GB RAM. Experiments were run in Jupyter notebooks [18] under Python 3.6. The scikit-learn package [19] was used for statistical analyses. Jupyter notebooks used for the experiments are available on GitHub [20] and include detailed information on hyperparamter sweeps and performance for each hyperparameter setting.

We investigated classification quality based on F1 scores weighted by the support of each label (i.e., how frequent each label occurred in the dataset). The scikit-learn package [19] was used for statistical analysis.

Since fastText is based on a relatively simple bag-of-N-grams model, it cannot utilize data on sentence sequences as-is. To utilize the sequential nature of sentences in PubMed abstracts, we devised a simple pre-processing step that gives fastText information about the position of a sentence in the abstract, as well as the content of preceding and following sentences in the abstract through additional tokens. These additional tokens are added to the sentence that is to be classified, and the standard bag-of-N-grams model of fastText is then trained on this enhanced sentence representation. Even though this increases the vocabulary of N-grams and the number of N-grams used to classify each sentence, fastText still remains highly performant. In pre-processing, the sentence representation was augmented by adding numeric sentence position information, as well as representations of the two preceding and trailing sentences. Tokens in these context sentences are altered by adding prefixes (e.g., ’-1_’ for the directly preceding sentence so that sentence sequence information is preserved in the fastText model). As an abstract example, the following represents a sequence of five sentences, with ‘aaa’, ‘bbb’ exemplifying tokens in each sentence:


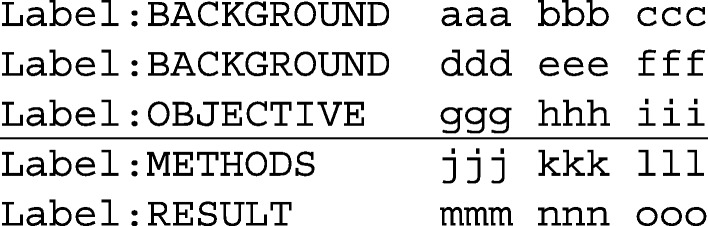


The preprocessing algorithm turned the third sentence (with the ‘objective’ label) into the following representation with additional tokens for training fastText (added tokens representing numeric sentence position and context sentences):


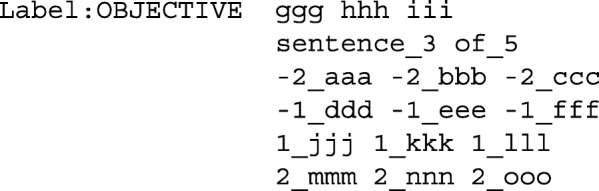


We also conducted ablation experiments where we removed parts of the preprocessing algorithm to quantify the benefit of each part of the algorithm.

#### Unsupervised model for sentence embeddings with sent2vec and fastText

sent2vec is an unsupervised model for learning universal sentence embeddings. It is an extension of the fixed-length word-contexts from CBOW to a larger sentence context of variable length. This extension allows for learning sentence embeddings in an additive manner by minimizing the unsupervised objective loss function (3). Thus, a sentence embedding **v**_*S*_ is generated by averaging the word (or n-gram) embeddings of its constituents.

$$\mathbf{v}_{S} := \frac{1}{|R(S)|} \mathbf{V} \mathbf{\iota}_{R(S)} = \frac{1}{|R(S)|} \sum\limits_{w \in R(S)} \mathbf{v}_{w}, $$

where *R*(*S*) is the list of n-grams (including unigrams) present in the sentence *S*. In the process of minimization we learn source **v**_*w*_ and target **u**_*w*_ embeddings for each word *w* in the vocabulary $\mathcal {V}$ as in (1). Similar to CBOW, sent2vec predicts a missing word from the context (which in the case of sent2vec is the entire sentence) via an objective function that models the softmax output approximated by negative sampling. Coupled with the logistic sigmoid function $l(x) = \log \left (\frac {1}{1 + e^{-x}} \right)$, the unsupervised training objective function is formulated as follows

3 $$ \underset{\mathbf{U}, \mathbf{V}}{\mathrm{arg\,min}} \sum\limits_{S \in \mathcal{C}} \sum\limits_{w_{t} \in S} \left(l\left(\mathbf{u_{w_{t}}}^{T} \mathbf{v}_{S \setminus {w_{t}}}\right) + \sum\limits_{w' \in N_{w_{t}}} l\left(\mathbf{u_{w'}}^{T} \mathbf{v}_{S \setminus {w_{t}}}\right) \right),  $$

where *S* corresponds to the current sentence and $N_{w_{t}}$ is the set of words sampled negatively for the word *w*_*t*_. This unsupervised model can also be used with fastText, however, the main difference will be in the definition of the context: fixed-length context for fastText and entire sentences (variable length context) for sent2vec. Detailed comparison and the evaluation of the two models can be found in [15].

To simulate settings in which limited training data were available, we created limited training datasets by randomly sampling sentences from the PubMed 200k training corpus. The number of sampled sentences for training was varied from 100 as the lowest to 180 000 as the highest sentence count, the latter being roughly equivalent to the number of sentences in the PubMed 20k training corpus. Three classifier setups were compared:

- *fastText:* The standard, fully supervised fastText algorithm
- *fastText, pre-trained:* A semi-supervised fastText model where N-gram embeddings were pretrained in an unsupervised way on the full PubMed 200k training text corpus (disregarding any labels) before switching to supervised training for sentence classification
- *sent2vec + multi-layer perceptron (MLP):* whole sentence embeddings were trained in an unsupervised way on the full PubMed 200k training corpus, vector representations of sentences generated by sent2vec were then used to train a multilayer perceptron with a single hidden layer (size 100 neurons) in a supervised way

Single sentences without sentence context or ordering were used for the evaluations of semi-supervised training. Hyperparameter settings for the fastText models were taken from the best-performing model established in the unsupervised task. The test set for each run consisted of 20 000 randomly sampled sentences that did not overlap with the training set sentences. For each training set size, each classifier was run on five random samples of training and test sentence sets, and the median weighted F1 scores on the test sets were calculated.

#### Code availability

Jupyter notebooks with code for training, testing and statistical analysis procedures, as well as trained models are available on GitHub [20].

## Results

### Fully supervised training

An overview of the results of our fastText models compared to other published results is shown in Table 2. The fastText model with sentence context and numeric sentence position provides a result for the PubMed 200k benchmark that outperforms the current state-of-the-art model by a small margin (F1 score of.917 vs. F1 score of.916 reported in [11] for the sophisticated bi-ANN deep learning model), while retaining a short training time of 73 s. For the smaller PubMed 20k corpus, fastText results are slightly worse than those of the bi-ANN model (.896 vs..900), while completing training in only 11 s. Expectedly, the fastText classifier based only on single sentences (without taking information on sentence sequence, sentence context or sentence position into account), yields lower F1 scores (.852 for PubMed 200k and.825 for PubMed 20k).

**Table 2:** Weighted F1 scores for various models trained on single sentences. Best results for each dataset are printed in bold. For our models, training time is given (for hyperparameter settings yielding the shown score)

Model	PubMed 20k	PubMed 200k	Extended corpus
Logistic regression model (LR) [11]^a^	.831	.859 (33,006 s)	-
Forward artificial neural network (ANN) [11]^a^	.861	.884	-
Conditional random field (CRF) [11]^a^	.895	.915 (4867 s)	-
bi-ANN [1]^a^	**.900**	.916	-
*fastText single-sentence (ours)*	.825 (5 s)	.852 (13 s)	.852 (61 s)
*fastText with sentence context and numeric sentence position (ours)*	.896 (11 s)	**.917 (73 s)**	**.919 (183 s)**

^a^Result and runtime reported by [11]; the reported runtimes given by authors include both training and testing time while we report only training time. Testing of a trained fastText model took approx. 15 s with the evaluation tool supplied by the fastText library.

fastText with sentence context and numeric text position trained on the extended corpus and evaluated on the PubMed 200k dataset achieves an F1 score of.919, showing that utilizing a larger training corpus can further improve classification quality. The extended training set size did not yield an improvement for the single-sentence fastText model.

Ablation experiments on the PubMed 200k benchmark showed that both the numeric sentence position and the addition of context sentences greatly benefitted classification quality, with the removal of context sentences yielding a greater degradation of quality than removal of numeric sentence positions (Table 3).

**Table 3:** Ablation experiments based on PubMed 200k dataset

	Weighted F1 score
Full model	.917
Removed numeric sentence position	.912
Removed sentence context	.904
Removed both sentence context and numeric sentence position (single sentence model)	.852

To further analyse the results of our best fastText classifier on the PubMed 200k dataset, we calculated the confusion matrix for the predictions on the test data split (Table 4). We found that while classification of methods, results and conclusion sentences is almost perfect, objective and background sentences are often mixed up. This problem has also been noted with the predictions of the deep bi-ANN model of [11].

**Table 4:** Confusion matrix for test results for the PubMed 200k dataset, yielded by the fastText model with sentence context and numeric sentence position

True label	Predicted label				
	Objective	Background	Methods	Results	Conclusions
Objective	**1704 (72%)**	573	98	0	2
Background	471	**2051 (77%)**	91	8	42
Methods	25	36	**9375 (96%)**	296	18
Results	3	3	395	**9744 (95%)**	131
Conclusions	1	23	13	203	**4186 (95%)**

### Semi-supervised training

**Fig. 2:**
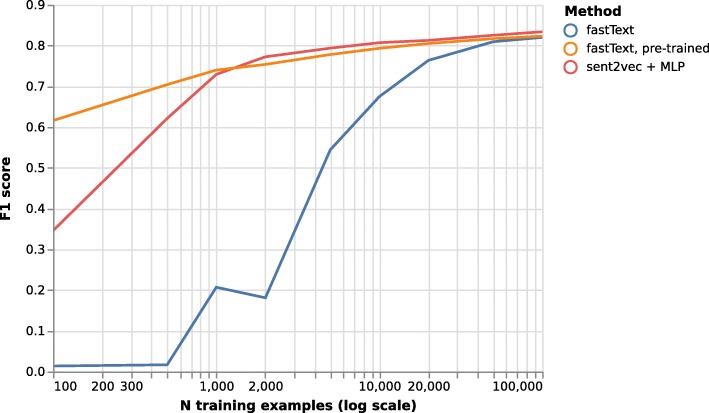
Weighted F1 score of test set predictions for different training set sizes

We found that the semi-supervised approaches and the fully unsupervised approach yield equal classification qualities for larger training set sizes of > 50000 training examples, where classification performance approached a ceiling with an F1 score of approximately 0.84 (Fig. 2). However, unsupervised pre-training yields a decisive advantage at smaller training set sizes. When training on a small training set of 1000 sentences, the fully unsupervised model did not yield useful results (weighted F1 of 0.21), while sent2vec+MLP and fastText with pre-trained word vectors yield far superior F1 scores (0.74 and 0.73, respectively). At even smaller training set sizes, fastText with pre-trained N-gram vectors was superior to sent2vec+MLP, while the two methodologies yielded similar results for training set sizes of 1000 sentences and above. Given these results and the greater ease of use of the methodology of using fastText with pre-trained, domain-specific N-gram vectors, this appears to be the methodology of choice.

## Discussion

fastText yielded good results with very low training times for both unsupervised and semi-supervised training. While we introduced a simple pre-proces5sing algorithm for utilizing sentence sequence information, it is noteworthy that fastText functioned well without much additional pre-processing (e.g., rare words and numbers were not removed from the corpora, resulting in a very high number of tokens fed into fastText that were not relevant to the classification task). Results were good across a wide variety of hyperparameter settings, demonstrating that the algorithm is quite robust. The main determinant of classification performance was the number of epochs, where, expectedly, low epoch numbers led to underfitting and higher numbers led to overfitting. Since fastText does not have an in-built methodology for early stopping, it is therefore indispensable to set up external scripts that do this hyperparameter optimization.

fastText requires little data pre-processing, little hyperparameter tuning, does not require a GPU, optional engineering of task-specific pre-processing steps is simple and intuitive, and training of models is very fast. We therefore suggest that fastText should be among the first methodologies to consider in biomedical text classification tasks. Overall, the robustness of results across a wide range of hyperparameter settings makes it possible to achieve good results with less hyperparameter tuning, which can further ease training when compared to more complex deep-learning methods that usually require extensive hyperparameter tuning.

The finding that the bag-of-N-grams model of fastText achieved similar classification quality as more detailed representations of sentence structure (e.g. precise word sequences as captured by recurrent neural networks) suggests that these detailed representations are not required for classification tasks. The bag-of-N-grams model captures enough of the “rough content” of a specific sentence, which seems to be sufficient for most classification tasks. Providing contextual information, such as the content of preceding and following sentences and the position of the sentence within the abstract improve classification quality, but can also also be captured through a bag-of-N-grams representation through the pre-processing algorithm we introduced.

How is fastText able to tackle difficult classification tasks with such short CPU training time? While other more complex neural network architectures capture word combinations and n-gram patterns through a cascade of recurrent or convolutional layers, fastText relies solely on scaling up the “width” of the shallow neural network, and on the distributional hypothesis of semantics of the words and n-grams within a given context. The neural embedding model includes two embedding layers, and the weight matrices of these embedding layers are the parameters of the model. The parameters are updated when the neural network is trained through the gradient descent-based optimization methods. At the end of training the columns of the weight matrix of the first embedding layer represent the final learned word embeddings (latent vector representations). The implementation of this tool is optimized to fast updates of the model parameters (i.e., embeddings of words and n-grams), in such a way that it scales very well for a very large number of tokens (“rows” in the weight matrix of the first embedding layer). While other models scale exponentially as we increase the number of tokens to capture the semantics of sentence embeddings, fastText scales linearly. Finally, provided that abstract texts use different n-gram patterns, for the different parts of the abstracts (e.g., conclusions, results), the classification task boils down to capturing the most salient features to discern these n-gram patterns. fastText is able to capture those features by increasing the width instead of the depth of its neural network architecture, which is why it is able to deal with the classification task on the subparts of the biomedical abstracts so quickly.

The ability of the fastText model to scale to very large vocabularies and n-grams can be used to represent more complex structures by simply representing them as additional entities that can be embedded. In the context of this work, we represented words in context sentences as separate entities, which multiplied the number of entities that are embedded, but was nonetheless easily handled by the algorithm.

### Limitations

A limitation of the fastText algorithm is that is not easily applicable to multi-label prediction (i.e., settings where a varying number of labels apply to one input text, instead of precisely one label per input text). fastText generates a single probability distribution over all labels with a softmax function (i.e. probability of all labels adding up to 1), which is not ideal if multiple labels can be correct for the same input text. While workarounds for this problem are available, it remains a limitation in use-cases that require multi-label prediction.

Another potential limitation of this work lies in the PubMed 200k RCT benchmark dataset. Both the models of [11] and our models have difficulty discerning sentences from the background and objective classes, and a sizable fraction of the difference between perfect F1 scores and observed F1 scores is caused by this difficulty. Reviewing a sample of abstracts in the dataset suggests that these classes are used in an inconsistent manner, and successfully discerning these classes might be difficult even for human expert annotators. This could imply that current best results of automated classifiers are already very close to the best possible scores that can be achieved, which would limit the utility of the benchmark dataset. Ideally, a gold-standard score based on the performance of expert human curators should be established for this benchmark.

### Future work

The presented methodology should be evaluated with other sentence classification use-cases and benchmarks. Further research should also be devoted to the exploration of semi-supervised approaches that combined unsupervised pre-training on large text corpora with supervised training on smaller corpora. Of special interest in this regard might be methods that are based on ensembles of different unsupervised sentence representations, such as an ensemble of the sent2vec model trained on single sentences with other models that work on sentence sequences, such as the deep Skip-Thoughts model [21]. The classifiers developed in this work could also be integrated into larger natural language processing pipelines for biomedical information extraction. For example, selecting only sentences that are classified as conclusion sentences might provide a better signal-to-noise ratio than using full abstracts for term co-occurrence analysis and other text extraction approaches. In terms of software for end-users (i.e., medical professionals and biomedical researchers) we plan to integrate the classifiers created in this work into a new version of the FindMeEvidence search system [22]. The goal of this search system is to provide users with a quick overview of the main findings of biomedical PubMed research articles. The classifier will be used to provide a condensed overview of the key findings of articles that matched a user query. It has also recently been demonstrated that fastText can provide competitive results at low training times when applied for link prediction in knowledge graphs [14], a domain that is fundamentally different from text classification. In future research we will further investigate if fastText can be successfully applied to more such types of data through preprocessing tricks similar to the ones we demonstrated in this paper.

## Conclusion

We demonstrated that through utilizing a simple preprocessing algorithm, the fastText model can provide state-of-the-art results in biomedical sentence classification at low computational cost. We characterized semi-supervised approaches based on neural embeddings that enable good classification results with a lower number of training examples compared to a fully supervised approach. We suggest that highly performant, shallow neural embedding models such as fastText should be among the first methodologies to be considered when classification needs to be made on data that can be represented as bags of tokens. We demonstrated that more structured data can be utilized through preprocessing. Future work should investigate the potential of this approach for a wide variety of data that go beyond simple text, such as structured knowledge graphs.

## Abbreviations

ANN: Artificial neural network
CBOW: Continuous bag-of-words
CRF: Conditional random field
GPU: Graphics processor unit
LSTM: Long short-term memory
LR: Logistic regression
MLP: Multi-layer perceptron
RCT: Randomized controlled trial
